# Experimental infection with equine herpesvirus type 1 (EHV-1) induces chorioretinal lesions

**DOI:** 10.1186/1297-9716-44-118

**Published:** 2013-12-05

**Authors:** Gisela Soboll Hussey, Lutz S Goehring, David P Lunn, Stephen B Hussey, Teng Huang, Nikolaus Osterrieder, Cynthia Powell, Jesse Hand, Carine Holz, Josh Slater

**Affiliations:** 1 Department of Pathobiology and Diagnostic Investigation, Michigan State University, East Lansing, MI 48824, USA; 2 Department of Clinical Sciences: College of Veterinary Medicine and Biomedical Sciences, Colorado State University, Fort Collins 80523, USA; 3 College of Veterinary Medicine, North Carolina State University, 1060 William Moore Dr, Raleigh, NC 27607, USA; 4 Institut für Virologie, Zentrum für Infektionsmedizin – Robert von Ostertag-Haus, Freie Universität Berlin, Robert-von-Ostertag-Str. 7-13, Berlin 14163, Germany; 5 Equine Referral Hospital, Royal Veterinary College, Hawkshead Lane, N. Mymms, Hatfield Herts AL9 7TA, UK

## Abstract

Equine herpesvirus myeloencephalitis (EHM) remains one of the most devastating manifestations of equine herpesvirus type 1 (EHV-1) infection but our understanding of its pathogenesis remains rudimentary, partly because of a lack of adequate experimental models. EHV-1 infection of the ocular vasculature may offer an alternative model as EHV-1-induced chorioretinopathy appears to occur in a significant number of horses, and the pathogenesis of EHM and ocular EHV-1 may be similar. To investigate the potential of ocular EHV-1 as a model for EHM, and to determine the frequency of ocular EHV-1, our goal was to study: (1) Dissemination of virus following acute infection, (2) Development and frequency of ocular lesions following infection, and (3) Utility of a GFP-expressing virus for localization of the virus in vivo. Viral antigen could be detected following acute infection in ocular tissues and the central nervous system (experiment 1). Furthermore, EHV-1 infection resulted in multifocal choroidal lesions in 90% (experiment 2) and 50% (experiment 3) of experimentally infected horses, however ocular lesions did not appear in vivo until between 3 weeks and 3 months post-infection*.* Taken together, the timing of the appearance of lesions and their ophthalmoscopic features suggest that their pathogenesis may involve ischemic injury to the chorioretina following viremic delivery of virus to the eye, mirroring the vascular events that result in EHM. In summary, we show that the frequency of ocular EHV-1 is 50-90% following experimental infection making this model attractive for testing future vaccines or therapeutics in an immunologically relevant age group.

## Introduction

Equine herpesvirus type 1 (EHV-1) infection is common in horses throughout the world and continues to cause significant economic losses through frequent outbreaks of a range of diseases including epidemic respiratory disease, abortion, neonatal foal death, equine herpesvirus myeloencephalopathy (EHM), and chorioretinopathy [1]. Primary infection with EHV-1 occurs via the respiratory tract and is followed by a cell-associated viremia, which is the prerequisite for infection of endothelial cells of the CNS, the pregnant uterus, or the eye. One of the most devastating manifestations of EHV-1 is EHM, which is the result of an inflammatory cascade that is associated with EHV-1 infection of the endothelial cells of the CNS. This infection results in damage to the microvasculature of the CNS due to microthrombosis, and extravasation of mononuclear cells causing perivascular cuffing and local hemorrhage [1,2].

Outbreaks of EHM are characterized by a large number of horses affected with mild to moderate respiratory disease and a fever, with up to 10% of infected horses developing EHM [3]. Neurological signs appear following the onset of viremia and include ataxia of the hind limbs that can lead to recumbency, loss of anal tone, flaccid paralysis of the tail and urinary incontinence often requiring euthanasia of the affected animal. However, despite the importance of EHM, we have a limited understanding of its pathogenesis, partly because of the lack of reliable and relevant models. Currently the only way to reproduce EHM experimentally in a significant proportion of animals is to perform challenge infections in horses over 20 years of age [4,5] (Dr Laura Maxwell, Oklahoma University, personal communication) with so called EHV-1 D752 strains*.* This “old horse” model can lead to clinical EHM in 50-70% of horses, but has several disadvantages. Firstly, horses of this age likely suffer from immunosenescence, making observations in this age group of dubious relevance to the horse population in general, [6,7] and making the model inappropriate for assessing immunological protection from EHM. Secondly, the generation of clinical EHM causes severe suffering in horses, typically leading to euthanasia and raising ethical concerns.

Further secondary manifestations of EHV-1 include late-term abortions and ocular disease [8], which result from infection of the endothelia of the pregnant uterus or the eye, respectively. Ocular EHV-1 may be of particular interest as a model for studying EHM pathogenesis because there is evidence that the vascular network of the equine ocular fundus is physiologically and anatomically similar to that of the CNS, with tight junctions analogous to the “blood brain barrier” [9]. In addition, EHV-1 induced chorioretinitis may be observed in a much larger percentage of horses than EHM, as one can observe subclinical infection in the eye which is not possible in the spinal cord [8]. EHV-1 associated chorioretinitis was first described in the late 1980’s in llamas and alpacas [10,11] and then in a mare and foal during a natural outbreak of paralytic EHV-1 infection [12]. In 1992, one of the authors showed that experimental infection with EHV-1 induced diffuse chorioretinopathies in a significant proportion of specific pathogen free (SPF) foals as well as endothelial infection of the ocular vascular endothelium, analogous to the known initial event in the pathogenesis of EHM where infection of spinal cord endothelial cells precipitates neurological disease [8]. In addition, in a series of experimental EHV-1 infections one of the authors (Slater) observed ocular lesions in approximately 50% of infected ponies (unpublished data), further supporting the need to investigate experimental induction of EHV-1 chorioretinopathy.

Based on these observations, the goal of the current study was to further elucidate the pathogenesis and course of EHV-1 induced chorioretinopathy, and to determine similarities to EHM pathogenesis. In addition, we wanted to determine the frequency with which EHV-1 induced chorioretinopathy occurs following experimental infection with the aim of using endothelial infection of the eye as an in vivo model for endothelial infection of the CNS. As a secondary objective, we investigated the use of fluorescein angiography (FA) in addition to classical fundus photography (FP) for detection of acute vascular lesions and delays in vascular filling time or leakage due to EHV-1 infection. Finally, a mutant virus expressing the enhanced green fluorescent protein (eGFP) was generated to allow for possible direct visualization of infected cells in the eye by fluorescent fundus photography (FFP).

## Materials and methods

### Animals

For experiment 1, 10 conventionally reared, seronegative ponies (SN < 1:32) were purchased and 8 ponies were infected with an Ab4 lac-Z mutant [13] designated Ab4Δ75-LacZ in this manuscript, while 2 ponies were infected with the parental Ab4 strain and served as controls. Ponies were group-housed in an outdoor pen with a walk-in shelter in Cambridge, UK, throughout the experiment, and were fed twice a day with a diet of hay and pelleted concentrate and *ad libitum* water.

For experiment 2, 12 EHV-1 seronegative horses (SN < 1:32), at an age of 9–18 months, were housed in a remote indoor facility in Fort Collins, CO throughout the experiment, and were fed twice a day with a diet of hay and pelleted concentrate and *ad libitum* water. All horses in this experiment were infected with EHV-1 strain Ab4.

For experiment 3, 12 EHV-1 seronegative horses (SN < 1:32), at an age of 9–18 months, were purchased and housed in a remote indoor facility in Fort Collins, CO, throughout the experiment. Horses were randomly assigned to two groups of 6 horses (Ab4 and Ab4GFP) and were fed twice a day with a diet of hay and pelleted concentrate and *ad libitum* water.

All experiments were performed in accordance with the Animal Care and Use Committee guidelines of Cambridge University (experiment 1) or Colorado State University (experiments 2 and 3).

### Generation and in vitro characterization of viruses

The neuropathogenic EHV-1 strain Ab4, a strain that is known to cause respiratory disease, abortions and neurological disease upon experimental inoculation, was used for all experiments as the parental virus [14]. In addition, two Ab4 mutants were generated for experiments 1 and 3. For experiment 1, an Ab4 mutant containing a LacZ gene was generated (Ab4Δ75-LacZ). In this virus, ORF75 was inactivated by replacing sequences between nucleotide position 136,115 and 113,8115 with the *E. coli* LacZ gene driven by the CMV IE promoter. The generation and characterization of Ab4Δ75-LacZ was previously described and a schematic of its genomic organization is given in Additional file 1a [13]. For experiment 3, Ab4 containing a GFP sequence inserted between ORF1 and ORF2 (Ab4GFP) was generated by two-step Red-mediated (*en passant*) recombination [15] using the mutagenesis primers Ab4GFP_FW: 5′ *CAAACGGCAAGAAGGAGAAATAAAACGACTGTAGTACCGC*GCGTTGACATTGATTATTGA 3′ and Ab4GFP_RV: 5′ *GAAAGGAGTGCATGTAAAAATAAATGCGATTAACCTTTGC*CATAGAGCCCACCGCAT3 (Additional file 1b). Briefly, to amplify the insertion cassette, a construct containing GFP-*aphAI* driven by the CMV promoter was created and used as template to yield the PCR product used for recombination [15]. The expression of gp2 by the recombinant virus was restored by co-transfection of pAb4_CMV_GFP BAC DNA and plasmid p71Ab4 into RK-13 cells, followed by several rounds of plaque purification to achieve a homogenous gp2-expressing population [16]. Before inoculation of ponies with Ab4GFP, replication and GFP expression of the Ab4GFP was tested in vitro in RK-13 cells and equine dermal cells (NBL6) grown in minimal essential medium (Sigma-Aldrich, St. Louis, MO, USA) supplemented with 10% fetal bovine serum (FBS), 100 U/mL penicillin and 0.1 mg/mL streptomycin (Sigma-Aldrich) in three independent experiments.

### Experimental design for in vivo studies

**Figure 1:**
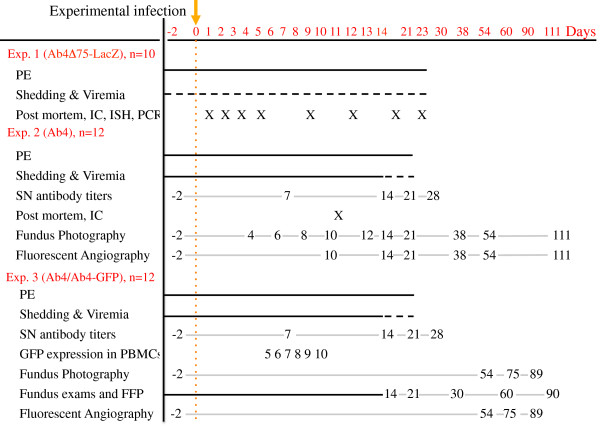
**Experimental design. Sampling regimen in the EHV-1 infection studies.** Experiment 1 consisted of ponies infected with Ab4 (*n* = 2) or Ab4Δ75-LacZ (*n* = 8), experiment 2 consisted of horses infected with Ab4 (*n* = 12), and experiment 3 consisted of horses infected with Ab4 (*n* = 6) or Ab4GFP (*n* = 6). Day 0 indicated by the dotted orange line was the time point of experimental infection. Continuous black lines indicate daily exams/sampling, dotted black lines indicate every other day sampling and continuous gray lines with numbers indicate specific sampling points. The X in experiments 1 and 2 indicate euthanasia and post mortem as well as immunohistochemistry (IC), in situ hybridization (ISH) and PCR where indicated.

Three separate experiments were performed in this study and a diagram of the experimental design is depicted in Figure 1. Experiment 1 was a pilot study investigating the pathogenesis and route of viral spread for ocular EHV-1 as well as viral distribution in the body during establishment of latency following intranasal infection. Two groups of conventionally reared ponies were infected with either the parental virus (Ab4), *n* = 2, or the Ab4Δ75-LacZ mutant containing a LacZ construct generated by Sun et al. [13], *n* = 8. Physical exams were performed daily from day -2 post-infection (pi) through day 23 pi and nasal swabs and PBMCs for detection of viral nasal shedding and viremia were collected on day -2 pi and every other day until day 23 pi. In the group of ponies infected with Ab4Δ75-LacZ, one pony each was euthanized on days 1, 2, 3, 5, 9, 12, 19 and 23 pi for post-mortem examination and detection of LacZ expression, EHV-1 gB DNA or LAT transcript in respiratory, lymphoid, ocular and neuronal tissues.

Based on the results in experiment 1 and previous unpublished data, a second experiment was performed in 12 horses to study frequency and ocular pathology following challenge infection with Ab4. For this purpose, clinical exams were evaluated immediately prior to and daily for 21 days following infection. Viral nasal shedding and viremia were evaluated daily for 14 days following infection, followed by every other day between days 14 and 21 pi. Serum for measurement of SN antibody titers was collected prior to infection and on days 7, 14, 21, and 28 pi. In addition, fundus exams and classical fundus photography (FP) were performed prior to infection and on days 4, 6, 8, 10, 12, 14, 21, 38, 54, 111 pi. Fluorescein angiography (FA) was performed prior to infection and on days 10, 14, 21, 38, 54, 111 pi.

In a third experiment, to further elucidate EHV-1 pathogenesis of the endothelium of the equine ocular fundus vascular network by fluorescent fundus photography (FFP), a GFP-expressing virus (Ab4GFP) was engineered and compared to infection with parental virus in two groups of 6 horses. Clinical exams were evaluated prior to infection and daily for 21 days pi. Nasal shedding and viremia were evaluated prior to infection and daily for 14 days pi, in addition to every other day between days 14 and 21 pi. Nasal swabs from all horses were also tested for GFP expression following infection, and GFP expression in PBMCs was evaluated on days 5, 6, 7, 8, 9 and 10 pi by flow cytometry. Serum for measurement of SN antibody titers was collected prior to infection and on days 7, 14, 21, and 28 pi. In addition, ocular exams and fluorescent fundus photography (FFP) were performed prior to infection and on days 1–14, 21, 30, 60, and 90 pi to directly detect virus expressing GFP. Finally, fundus photography (FP) as well as fluorescein angiography (FA) were performed prior to infection, and on days 54, 75 and 89 pi in order to highlight retinal pigmented epithelial defects secondary to EHV-1-induced chorioretinopathy (FA).

### In vivo characterization of viruses

#### EHV-1 infections

In experiment 1, ponies in both groups were simultaneously infected with 5 × 10^7^ PFU of the respective viruses in a total volume of 2 mL administered (1 mL per nostril) via intranasal instillation as described previously [17]. For experiments 2 and 3, horses were infected with 5 × 10^7^ PFU of EHV-1 strain Ab4 or Ab4GFP in a total volume of 10 ml administered via nasopharyngeal instillation and an aerosol was produced by inserting a plastic nozzle attached to a syringe into the nostrils as described previously [18].

#### Clinical monitoring, blood and nasal swab collection

For all three experiments, physical exams were performed by the attending veterinarian and rectal temperatures and clinical signs were recorded, including nasal or ocular discharge, coughing, malaise, inappetence and neurological signs.

In Experiment 1, nasal mucus for detection of virus shedding was collected by aspiration using a vacuum pump, as previously described [19]. In Experiments 2 and 3, nasal swabs for detection of EHV-1 viral shedding were collected using a sterile, 6-inch DACRON swab (Fisher Scientific Inc. Pittsburgh, PA, USA) as previously described [20]. The swab was inserted into one nostril and then the tip was placed in 1 mL virus isolation transport media (MEM with Gentamicin and Amphotericin B).

Blood for detection of viremia was collected in all three experiments as indicated in the experimental design section and Figure 1. Blood was collected by jugular venipuncture into heparinized tubes in all instances, and PBMC were separated as previously described [20]. In addition, in experiment 3, blood was collected into heparin on days 5 through 10 to determine GFP expression in PBMCs. For this purpose, PBMCs were isolated as previously described [20] and GFP expression was evaluated using flow cytometry.

#### Detection of viral nasal shedding and viremia

For experiment 1, virus shedding in nasal secretions and cell associated viremia was determined using classical plaque and infectious center assays as described previously [19]. For experiments 2 and 3, viral DNA in nasal swab samples or from PBMC was isolated using the QiaAmp DNA blood mini kit, according to manufacturer’s instructions (Qiagen, Inc., Valencia, CA, USA). To determine nasal virus shedding, viral DNA load was determined by real-time PCR using primers and a specific probe targeting glycoprotein B (gB) as previously described [21]. β-actin was used as the cellular housekeeping gene using primers and probe as developed previously [22]. Viral load was expressed as the log of EHV-1 gB DNA copies / 10^6^ β-actin copies [20].

Nasal swabs from all 12 horses from experiment 3 on day 2 pi were also cultured using RK-13 cells. Briefly, 250 μL of nasal swab samples were added to 90% confluent RK-13 and the virus was allowed to adhere for 2 h. After 2 h, the virus containing media was aspirated and replaced with 5 mL of Minimal Essential Medium (Invitrogen) containing 2% FBS. Cells were incubated for 72 h and checked daily for signs of CPE and GFP expression using regular and fluorescence microscopy.

#### Serum neutralization titers

Serum neutralization (SN) titers for experiments 2 and 3 were determined on days -1, 7, 14 and 21 pi as previously described [23].

### Necropsy and tissue collection

In experiment 1, ponies were humanely euthanized by intravenous injection of sodium pentobarbital at 85.8 mg/kg BWT at the times indicated in the experimental design section (Figure 1) and respiratory (nasal turbinate, trachea, lung), lymphoid (submandibular, retropharyngeal, tracheobronchial and mediastinal lymph nodes), ocular (cornea, aqueous, iris, vitreous, choroid, retina and optic nerve) and nervous system (trigeminal ganglion, medulla, pons, midbrain, optic nerve, forebrain and cerebral cortex including the olfactory lobes) tissues were collected. Samples were divided and snap frozen in liquid nitrogen for later DNA extraction or fixed in ice-cold 5% paraformaldehyde for 24 h before routine wax embedding and histological processing. In experiment 2, horse 11 was euthanized following development of EHM, and spinal cord and ocular tissues were collected into 5% paraformaldehyde for 24 h before routine wax embedding and histological processing as described above.

### Detection of EHV-1 gB DNA by nested PCR

In experiment 1 only, nested PCR analysis was conducted on DNA extracted from frozen tissues using a commercial kit (DNeasy mini kit, Qiagen) following the manufacturer’s instructions. Detection of EHV-1 DNA was carried out using a gel-based qualitative PCR method detecting a fragment of the EHV-1 gB gene, as previously described [24].

### Histopathology, detection of LacZ expression and in situ hybridization

In experiment 1 only, for detection of beta galactosidase activity, 10 μm sections were cut and stained with X-gal (5 bromo-4-chloro-3-indolyl β-D-galactoside) using standard methods [25] and then counterstained using haematoxylin and eosin. EHV-1 DNA (gB gene) and latency-associated transcripts (LAT) transcripts were detected by in situ hybridization carried out on histology sections of tissues as previously described [26], using digoxigenin-labeled DNA probes generated either from the nested PCR amplicon of the gB gene of EHV-1 or from the *Bam*HI fragment of EHV-1 to detect RNA antisense to ORF63.

### Ocular fundus photography (FP) and fluorescein angiography (FA) and fluorescent fundus photography (FFP)

Ocular fundus exams, fundus photography (FP), and fluorescein angiography (FA), were performed in experiments 2 and 3 following sedation with detomidine (0.02-0.04 mg/kg, i.v.) and dilation of the pupil with 2 drops of tropicamide 1%, and phenylephrine 2% (Bausch&Lomb Inc.,Tampa, FL, USA). For the FA, 2–3 mL of 25% Na-fluorescein was injected into the jugular vein (2 mg/kg) and FA of the left eye was immediately performed using a scanning Digital Ophthalmoscope (Wild MedTec, Switzerland) for 90 s. For detection of GFP in the eye by FFP, the same hand-held digital fundus ophthalmoscope and filter were used without injection of Na-fluorescein. Because all lesions showed a similar phenotype appearing as classical donut-shaped depigmented lesions with pigmented centers (focal or multifocal bullet or shotgun lesions) and differences were most notable in the number of lesions occurring in different horses, a scoring system based on number of lesions per eye was implemented. For this purpose, grade 1 equaled 1–3 lesions, grade 2 equaled 4–10 lesions, and grade 3 equaled 11 or more lesions throughout the non-tapetal fundus.

### Statistical analysis

In experiment 3, unpaired t-tests with Welch correction were used to determine differences in clinical scores, primary and secondary fever responses, nasal viral shedding and viremia between Ab4- and Ab4GFP-infected horses and a *p*-value < 0.05 was considered significant.

## Results

### In vitro characterization of mutant viruses

The in vitro characterization of the Ab4Δ75-LacZ is described elsewhere [13]. Growth kinetics of Ab4 and Ab4GFP were compared using RK-13 (Additional file 2) and NBL6 cells (not shown). Measurement of both intracellular and extracellular virus titers showed that the insertion of the GFP gene did not alter in vitro growth kinetics of Ab4GFP when compared to Ab4.

### In vivo characterization of viruses

**Figure 2:**
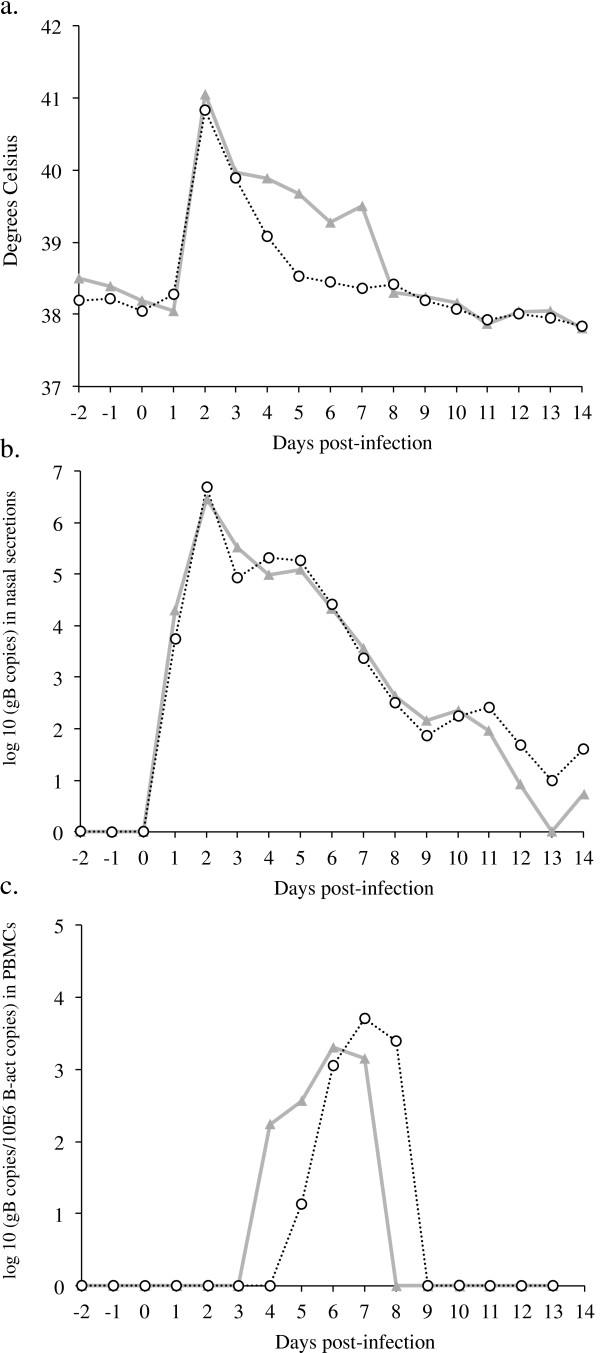
**Body temperature, nasal shedding and viremia following experimental EHV-1 infection in experiment 3. A**. Median body temperature expressed in °C, **B**. Median nasal viral shedding in nasal secretions expressed as EHV-1 gB copies, **C**. Median viremia in PBMCs expressed as log gB copies/10^6^ β-actin copies. Horses infected with the parental virus strain Ab4 (*n* = 6): -∆-, horses infected with Ab4GFP (*n* = 6): -Ο-.

Horses infected in all three experiments developed the classical signs of EHV-1 respiratory disease, including biphasic fever, nasal and ocular discharge, depression and coughing. Clinical signs were similar between the parental virus (Ab4) infected ponies and the ponies infected with the Ab4Δ75-LacZ mutant in experiment 1, but a statistical comparison was not done in this pilot experiment because of different group sizes and the decrease in size of the Ab4Δ75-CMV-infected group over successive time points. There were no significant differences in experiment 3 between the parental virus and the mutant virus except for the significantly lower secondary fever response in horses infected with Ab4GFP (*p* = 0.015) (Figure 2a).

As reported above, one horse in experiment 2 (#11) developed EHM (grade 5/5) [27] with paralysis in both hind limbs on day 10 pi and required euthanasia. None of the other horses in any experiment showed any neurological disease.

Similarly, as is typical for experimental EHV-1 infection, animals in all three experiments shed virus for multiple days following experimental infection and all horses, except for one horse in the Ab4 infection group of experiment 3 that never showed viremia, were viremic for a total duration of 1–7 days between days 3 and 9 following infection. Nasal shedding did not differ between parental viruses and mutant viruses in experiment 1 (data not shown) or experiment 3 (Figure 2b). In experiment 3, horses infected with the Ab4GFP virus showed a slightly delayed onset of viremia between days 4 and 9 when compared to horses in the Ab4 infection group that showed viremia between days 3 and 8 post-inoculation (Figure 2c).

Expression of GFP was detected following inoculation of RK-13 cells with nasal swabs from horses infected with the Ab4GFP virus (data not shown). In contrast, horses of experiment 3 that were infected with Ab4, showed CPE only following inoculation of RK-13, but no GFP expression was observed at any time (data not shown). Expression of GFP in PBMCs collected from Ab4GFP infected horses could not be detected when counting a total of 10^6^ cells using flow cytometry on days 5–10 pi (data not shown).

The SN titers in all horses from experiments 2 and 3 are shown in Table 1. Prior to inoculation with EHV-1, all horses from experiments 2 and 3 had EHV-1 SN titers below 1:32. After challenge infection, all horses seroconverted, with titers ranging from 1:128–1:2,048 by day 21 post-challenge.

**Table 1:** Serum neutralization titers in horses from the Ab4 infection (experiment 2) and the Ab4 vs. Ab4GFP infection (experiment 3).

**SERUM NEUTRALIZATION TITERS**				
**Ab4 infection (Exp.2)**				
Horse	Day -1	Day 7	Day 14	Day 21
1	2	64	1024	1024
2	8	64	2048	2048
3	4	128	1024	1024
4	8	64	1024	1024
5	2	32	512	512
6	2	32	256	256
7	8	32	256	1024
8	8	64	1024	2048
9	4	64	1024	1024
10	8	64	1024	1024
11	8	32	n.d.	n.d.
12	32	64	1024	2048
**Ab4 vs. Ab4GFP infection (Exp.3)**				
Horse	Day -1	Day 7	Day 14	Day 21
A (Ab4GFP)	2	2	128	256
B (Ab4)	2	4	64	256
C (Ab4GFP)	2	4	128	512
D (Ab4GFP)	2	2	32	128
E (Ab4GFP)	2	4	32	256
F (Ab4GFP)	2	4	16	128
G (Ab4)	2	8	64	128
H (Ab4)	2	8	256	512
I (Ab4)	4	8	512	512
J (Ab4)	2	4	64	128
K (Ab4GFP)	2	4	128	256
L (Ab4)	4	8	128	128

### Detection of Ab4Δ75-LacZ and LAT transcript by in situ hybridization (experiment 1)

**Figure 3:**
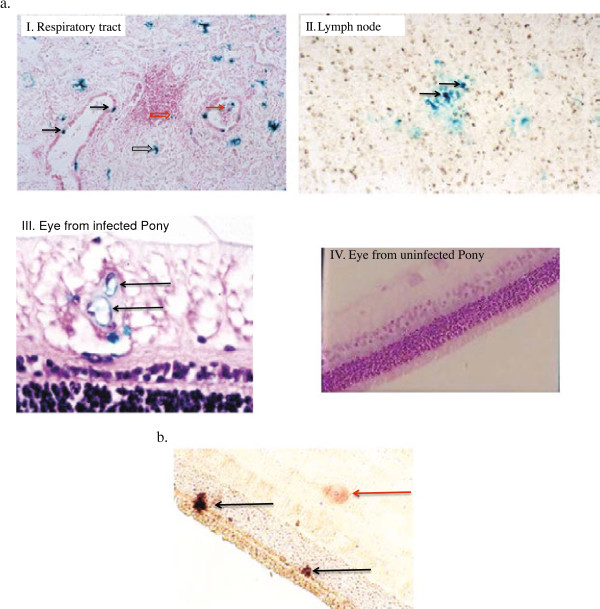
**Expression of LacZ indicating the presence of AB4Δ75-LacZ or EHV-1 gB DNA expression following experimental infection. A**. LacZ expression as indicated by X-gal positive cells representing presence of AB4∆75-LacZ. I. Respiratory tract 24 h pi: Beta galactosidase positive bronchial epithelial cells (black solid arrows); positive airway debris (red solid arrow); positive interstitial cells (black open arrows). A positive mononuclear cell, located within a perivascular cuff, is shown by a red open arrow. H&E stain, magnification × 50. II. Section of submandibular lymph node 48 h after infection with AB4∆75-LacZ showing beta galactosidase positive mononuclear cells (black solid arrows). H&E stain, magnification × 400. III. Section of neurosensory retina 9 days after infection with AB4∆75-LacZ showing beta galactosidase positive endothelial cells (black solid arrows). H&E stain, magnification × 400. IV. Section of neurosensory retina from an uninfected pony stained with X-gal and H&E counter stain, magnification × 200. **B**. In situ hybridization showing transcription of the late gB gene DNA in photoreceptors of the neurosensory retina on day 12 pi. Positive photoreceptor nuclei (black solid arrows) and a single optic nerve ganglion cell nucleus (solid red arrow) are shown. No counter stain, magnification x 200.

**Figure 4:**
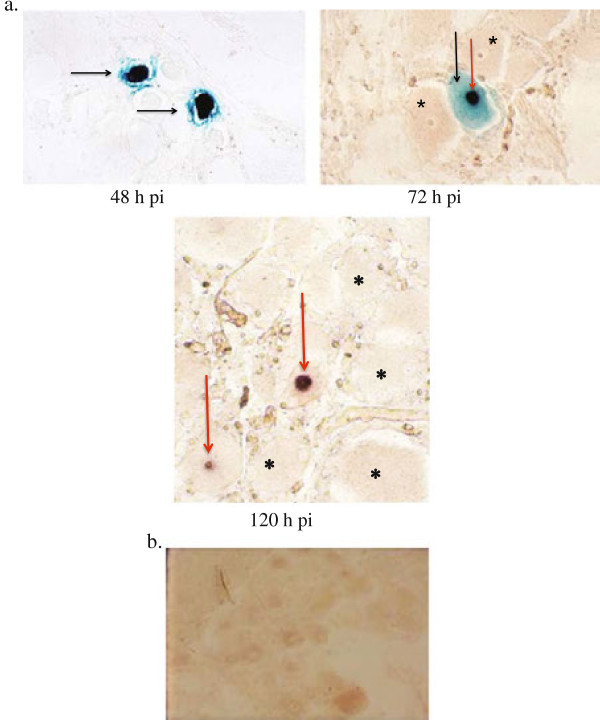
**Detection of AB4Δ75-LacZ and expression of latency associated transcripts (LAT) in trigeminal ganglionic neurons. A**. LacZ expression as indicated by X-gal positive cell nuclei representing presence of AB4∆75-LacZ at 48 h and 72 h pi. Two ganglionic neurons with nuclear localization of beta galactosidase activity are shown at 48 h pi (solid arrows). No counter stain, magnification × 400. At 72 h pi, one ganglionic neuron with nuclear localization of beta galactosidase activity (black arrow) and nucleolar localization of LAT (red arrow) is shown. Adjacent neuronal nuclei are negative by both techniques (*). No counter stain, magnification × 400. At 120 h pi, nucleolar expression of LAT only is shown in two ganglionic neurones with nucleolar localization of LAT (red arrow). The nuclei are negative for beta galactosidase activity. Adjacent neuronal nuclei are negative by both techniques (*). No counter stain, magnification × 400. **B**. No detection of LacZ expression as indicated by X-gal positive cell nuclei or LAT expression in the trigeminal ganglion from an uninfected control pony. No counter stain, magnification × 400.

LacZ expression could clearly be detected in the respiratory tract on day 1 (airway epithelial cells, intraluminal debris, interstitial cells and mononuclear cells associated with perivascular cuffs) and the mononuclear cells in lymph nodes draining the respiratory tract on days 2 and 3 pi (Additional file 3 and Figure 3a). In addition, LacZ expression was detected in endothelial cells of the retina and the choroid on day 9 pi (Additional file 3 and Figure 3a), but not in the photoreceptors of the retina (Additional file 3 and Figure 3a). In contrast, LacZ expression was detected in the nuclei of neurons of the trigeminal ganglion at 48 and 72 h pi, but expression was no longer detectable by 120 h pi (Additional file 3 and Figure 4a). Interestingly by 72 h pi, some trigeminal ganglionic neurons presented with LacZ expression in the nucleus and were also positive for LAT expression in the nucleolus (Figure 4a). By 120 h pi, there were no LacZ-positive neurons detectable, while some neurons were LAT-positive (Figure 4a). No LacZ expression could be detected at any time in the medulla, pons, tectum, tegmentum, rostral colliculus, lateral geniculus, optic chiasm, optic nerves and olfactory lobes (data not shown). LacZ or LAT expression was also not detected in neuronal tissues of an uninfected control pony (Figure 4b).

### Detection of EHV-1 gB DNA by nested PCR and by in situ hybridization (experiment 1)

Retinal tissues were positive for gB DNA by nested PCR on days 9, 12, 19 and 23 pi and choroid samples were positive on day 12 pi (Table 2). In addition, occasional photoreceptors were positive for gB DNA by in situ hybridization on day 12 (Figure 3b). Interestingly, while no virus-positive cells were detected by LacZ expression or in situ hybridization in any of the CNS tissues (data not shown), EHV- gB DNA was detected in the, medulla, pons, midbrain, optic nerve, forebrain and cerebral cortex including the olfactory lobes by PCR between days 3 and 23, but in particular on days 9, 12, 19 and 23 (Table 2). In addition EHV- gB DNA was detected in the trigeminal ganglion at all time points studied (Table 2).

**Table 2:** Detection of EHV-1 gB DNA by nested gB PCR in ocular and nervous system tissues collected post mortem following infection of ponies with Ab4∆75-LacZ (experiment 1).

**Pony #**		**A**	**B**	**C**	**D**	**E**	**F**	**G**	**H**
**Tissue**	**Day pi/euthanasia**	**1**	**2**	**3**	**5**	**9**	**12**	**19**	**23**
Cornea		+	+	-	+	-	+	+	-
Aqueous		-	+	+	-	+	-	-	-
Vitreous		-	-	-	+	+	+	-	-
Iris		-	-	-	-	+	-	-	-
Choroid		-	-	-	-	-	+	-	-
Retina		-	-	-	-	+	+	+	+
Optic nerve		-	-	-	-	+	-	+	+
Olfactory lobe		-	-	-	-	+		+	-
Cerebrum (frontal)		-	-	-	-	+	-	-	+
Cerebrum (parietal)		-	-	-	+	-	-	+	-
Cerebrum (occipital)		-	-	-	-	+	-	-	-
Cerebrum (temporal)		-	-	-	-	-	+	+	-
Choroid plexus		-	-	-	+	+	+	-	+
Diencephalon		-	-	-	-	+	-	+	+
Midbrain		-	-	+	-	+	+	-	-
Pons		-	-	-	-	+	+	-	+
Medulla		-	-	-	-	+	+	-	+
Cerebellum		-	-	-	-	-	+	-	-
Trigeminal ganglion		+	+	+	+	+	+	+	+

+ detection of EHV-1 gB DNA; - no detection of EHV-1 gB DNA.

### Ocular fundus exams and fluorescent angiography

**Figure 5:**
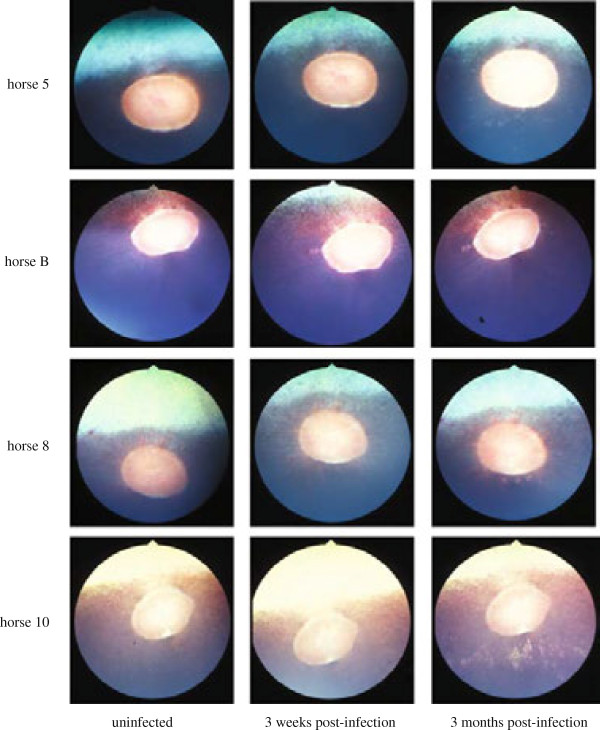
**Development of ocular lesions following infection with EHV-1 shown by fundus photography (FP).** FP in 4 representative horses from experiments 2 and 3 prior to infection, as well as 3 weeks and 3 months after infection.

**Figure 6:**
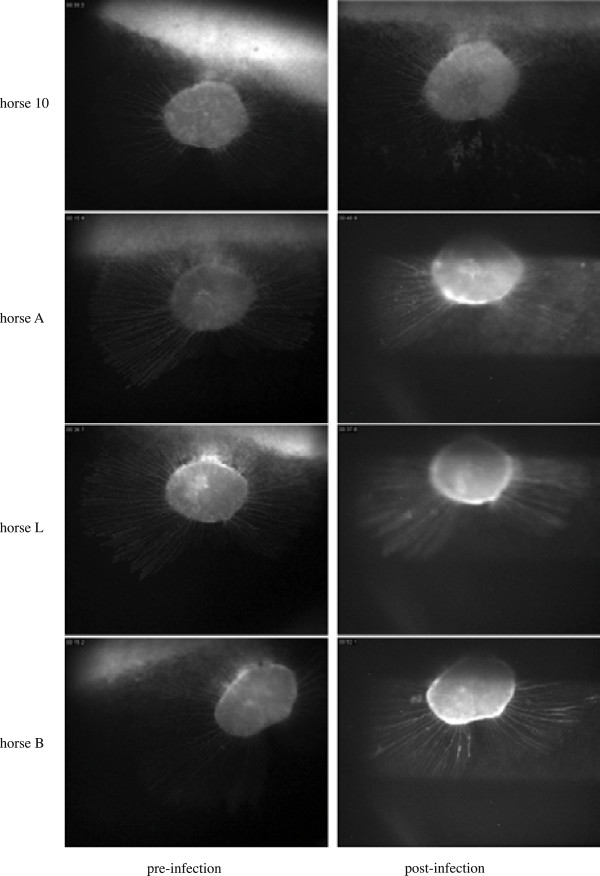
**Development of ocular lesions following infection with EHV-1 as shown by fluorescent angiography (FA).** FA images from 4 representative horses from experiments 2 and 3 prior to infection and 3 months after infection.

In experiment 1, no ocular clinical signs were detected and none of the ponies developed ophthalmoscopically visible chorioretinal lesions in the short period before euthanasia. In experiment 2, no ocular clinical signs were detected, but nine out of 12 horses developed ophthalmoscopically visible chorioretinal lesions between days 20 and 111 pi (Table 3, Figures 5 and 6). These lesions were multifocal, small, and irregularly circular lesions that occurred close to the optic disc usually, but not exclusively, within the limits of the retinal vasculature. The lesions presented as classical donut-shaped, depigmented lesions of the chorioretina with pigmented centers (also referred to as “focal” or “multifocal bullet” or “shotgun” lesions [8,9]). There was no evidence of attenuation of retinal vasculature in regions where lesions and retinal vessels were co-located (Figures 5 and 6).

**Table 3:** Ocular lesions in experiment 2: Ab4 infection.

**ID #**		**Pre-CH**	**Day 10**	**Day 14**	**Day 20**	**Day 38**	**Day 54**	**Day 111**	**New lesions post-infection**
**1**	OS	-	X	-	-	-	-	-	
	OD	-	X	-	-	-	-	-	
**2**	OS	-	X	-	-	-	1	X	*
	OD	-	X	-	-	-	1	X	*
**3**	OS	Walleye	X	X	X	X	X	X	
	OD	Walleye	X	X	X	X	X	X	
**4**	OS	-	X	-	-	-	2	3	*
	OD	-	X	-	-	-	1	1	*
**5**	OS	2	X	2	X	2	3	3	*
	OD	-	X	-	1	1	X	3	*
**6**	OS	2	X	2	2	2	3	X	*
	OD	1	X	1	1	2	2	2	*
**7**	OS	1	X	1	1	2	2	3	*
	OD	1	X	1	1	2	X	2	*
**8**	OS	-	X	-	-	-	1	1	*
	OD	-	X	-	-	-	-	2	*
**9**	OS	1	X	1	2	X	3	X	*
	OD	-	X	-	-	-	3	X	*
**10**	OS	-	X	-	-	-	3	X	*
	OD	-	X	-	-	-	3	X	*
**11**	OS	-	(EHM)	X	X	X	X	X	
	OD	-	(EHM)	X	X	X	X	X	
**12**	OS	1	X	1	X	1	1	2	*
	OD	-	X	-	-	-	-	-	

OS: left eye, OD: right eye, X: no ocular exam performed, *: new lesions post-infection, -: no lesions, 1: 1–3 lesions, 2: 4–10 lesions and 3: 11 or more lesions.

One of the 12 horses was a “walleye” characterized by a lack of pigment in the retinal pigmented epithelium. This horse and its ocular data was not considered for the study, as detection of chorioretinal lesions of the type previously reported for EHV-1 infection would have been difficult in a color-dilute fundus. Horse #11 developed EHM on day 10 pi and had to be euthanized. No ocular lesions were detected at this time by FP or FA. While no ocular lesions were found in vivo on day 10 pi, EHV-1 viral antigen was detected in both the eye and the spinal cord of this horse post mortem using immunohistochemistry and an EHV-1 specific antibody (data not shown).

Fluorescent angiography delineated the lesions visible by classical fundus photography with improved clarity and suggested that these lesions were likely caused by focal loss of retinal pigmented epithelium with exposure of underlying choroid, because fluorescing choroid was visible within lesions (Figure 6). No fluorescein leakage was observed from either retinal or choroidal blood vessels suggesting that there was integrity of the vascular walls, although choroidal vessels were unlikely to be visible prior to disruption of the RPE. Fluorescent angiography confirmed that there was no attenuation of the retinal vasculature in eyes with chorioretinal lesions (Figure 6).

In experiment 3, three of six horses in each group developed new ocular lesions starting approximately 3 weeks pi (Table 4). In the Ab4GFP group, no GFP-expressing cells were detected in vivo at any point using the scanning Digital Ophthalmoscope (Wild MedTec, Switzerland) and a filter capable of detecting GFP protein.

**Table 4:** Ocular lesions in experiment 3: Ab4 vs. Ab4GFP infection.

**ID #**	**Virus used**		**Pre-CH**	**Day 54**	**Day 75**	**Day 89**	**new lesionspi**
**A**	Ab4GFP	OS	-	-	X	1	*
		OD	-	-	X	1	*
**B**	Ab4	OS	-	X	2	2	*
		OD	-	1	3	3	*
**C**	Ab4GFP	OS	-	-	-	1	*
		OD	1	-	-	1	
**D**	Ab4GFP	OS	2	-	-	2	
		OD	-	-	-	-	
**E**	Ab4GFP	OS	-	-	-	1	*
		OD	-	-	-	1	*
**F**	Ab4GFP	OS	-	-	X	-	
		OD	-	-	X	-	
**G**	Ab4	OS	-	-	X	-	
		OD	2	-	X	2	
**H**	Ab4	OS	-	-	-	-	
		OD	-	-	-	-	
**I**	Ab4	OS	-	-	-	1	*
		OD	-	-	-	-	
**J**	Ab4	OS	-	-	X	-	
		OD	1	-	X	1	
**K**	Ab4GFP	OS	-	-	X	-	
		OD	1	-	X	1	
**L**	Ab4	OS	-	1	X	1	*
		OD	1	-	X	1	

OS: left eye, OD: right eye, X: no ocular exam performed, *: new lesions post-infection (pi), -: no lesions, 1: 1–3 lesions, 2: 4–10 lesions and 3: 11 or more lesions.

## Discussion

While it has been known for more than 25 years that EHV-1 infection leads to chorioretinopathy causing permanent focal or multifocal “shotgun” lesions of the chorioretina in a substantial proportion of infected horses, [8,9], this study is the first to evaluate the frequency of ocular EHV-1 following experimental infection with a neuropathogenic strain of EHV-1. We show in separate experiments that between 50% and 90% of experimentally infected horses develop chorioretinal lesions. Interestingly, clinically-apparent acute ocular pathology was not observed in vivo during the period of acute infection highlighted by biphasic fever responses and viremia, and horses showed no clinical signs of ocular disease. Specifically there were no signs of corneal disease or of uveitis and the later appearance of chorioretinal lesions was not associated with a loss of vision or any clinical ocular signs. However, ocular lesions developed between weeks 3 and 7 bilaterally or unilaterally and were visible both by FP and FA. The ophthalmoscopic appearance of the lesions did not change after first detection suggesting that they were static, possibly representing permanent pathological changes in the chorioretina in response to an insult occurring following EHV-1 infection. Acute ocular and spinal cord pathology and viral antigen could only be detected at *post mortem* in the one horse that was euthanized due to severe EHM on day 10 pi despite the absence of ocular findings *ante mortem*.

Horses in our experiments were not studied beyond the times indicated in the individual experiments and no long-term data were collected. This type of data may however be important to gather in future experiments, as long-term studies evaluating progression or, alternatively, resolution of ocular lesions following EHV-1 infection will be important for improved assessment of significance of ocular lesions and reliable prognosis.

Historically, chorioretinal lesions have been divided into three categories including, focal “bullet” lesions, multifocal “shotgun” lesions and diffuse lesions [9]. Following experimental EHV-1 infection, we observed both focal and multifocal lesions that were either unilateral or bilateral and located in the non-tapetal fundus as donut-shaped depigmented lesions with pigmented centers. This type of lesion is typically caused by endothelial damage with subsequent ischemic injury to the chorioretina that could result from direct infection of the vascular endothelium following viremia [28]. The absence of acute ocular clinical signs does not suggest ascending infection from an initial keratitis or uveitis as potential causes of the lesions observed. However, the appearance of lesions after the viremic phase of infection would be consistent with ischemic episodes within the choroidal microcirculation resulting in focal death of overlying retinal pigment epithelium and possibly regions of the neuroretina. Furthermore, the data from experiment 1, although preliminary in nature, do not suggest that EHV-1 translocates to the eye by direct extension through the central nervous system using the olfactory lobes of trigeminal tracts as portals of entry. The data presented here provides preliminary evidence that the pathogenesis of EHV-1-induced chorioretinopathy may mirror the vasculopathy seen in uterine and CNS vessels during abortion and EHM. Therefore, it may be possible that the vascular network in the fundus of the eye can be used to monitor EHV-1-induced endothelial vasculopathy and serve as “a window into the microcirculation of the infected animal”.

Taken together, the observations from our study suggest that ocular lesions following EHV-1 infection are caused by a focal loss of retinal pigmented epithelium (RPE) exposing the underlying choroid. The apparent lack of attenuation of the retinal circulation suggests that disruption to the neuroretina is confined to the outer layers (photoreceptors and/or bipolar cells) in some or all of these lesions. However, detailed histopathological examination of affected areas of the chorioretina is required to further define the lesions and this work is on-going in our laboratory. The lack of attenuation of retinal vasculature is particularly interesting because the major blood supply to the neuroretina in the horse, in contrast that in other species, is provided by the underlying choroidal vessels whereas the retinal vasculature is limited [9,29-31]. If EHV-1 induced vascular injury in fact occurred mainly in the choroidal vasculature, it may explain why lesions are not visible during the early stages of pathogenesis but only become detectable ophthalmoscopically once ischemic death of the RPE leads to focal breaks, thus exposing the underlying choroid, which happens some time after the initial ischemic episode. Further supportive of the interpretation that the virus reaches vascular endothelia of the eye via the blood is data obtained in experiment 1, which shows replicating virus, as measured by LacZ expression, in the respiratory tract, lymphocytes as well as the choroidal and retinal endothelium, while it is clearly absent from CNS tissues.

Horses in all three experiments developed the classical signs of EHV-1 respiratory disease and there were no significant differences in experiments 1 or 3 between parental viruses and mutant viruses, except for the significantly lower secondary fever response in horses infected with Ab4GFP in experiment 3. While the same virus strain, viral dose and experimental conditions were used for the parental virus groups in experiments 2 and 3, differences in duration of viral nasal shedding, magnitude of SN titers pi, and incidence of ocular lesions could be observed between Ab4-infected groups in the separate experiments. This implies that, in addition to viral factors including DNA polymerase [32,33], host and possibly environmental factors are likely to play a substantial role in the pathogenesis of EHV-1 infection and endotheliotropism, similar to what has been reported for the risk of developing EHM [34].

The Ab4GFP-expressing virus was designed with the aim of directly visualizing the virus in the eye during the acute phase of infection. Despite clearly demonstrable in vitro GFP expression following infection of equine cells, and recovery from the nasal secretion of virus expressing GFP, GFP expression following Ab4GFP infection could not be detected in the eyes of any horse at any time pi. Similarly, PBMCs isolated daily during the phase of viremia did not allow for detection of GFP expression at any time using direct flow cytometric analysis. Reasons for this surprising result may be technical limitations as well as numbers of cells actually infected with virus (i.e. only 1 in 10 000 PBMCs infected), as it is possible that insufficient numbers of GFP-expressing cells were present to be detectable by fundus photography or flow cytometry. It is also conceivable that the localization of the virus in the choroidal vasculature, which is hidden by the RPE, prevents visualization of GFP- expressing cells.

The use of fluorescein angiography did not offer advantages in the detection of ocular lesions compared to conventional fundus photography but did provide valuable insight into the possible pathological changes present within lesions. The fact that no differences in filling time or leakage of fluorescein were observed during the acute stages of infection suggested that inflammatory processes are unlikely to occur in the retinal circulation, which is the only circulation that can be observed during the acute phase of infection.

In summary, this study showed that the frequency of ocular lesions induced by experimental infection with EHV-1 occurs in a large proportion of animals (50-90%). This percentage is significantly higher then the incidence of naturally occurring EHM or EHM frequency in experimentally infected young or middle-aged horses. It is likely that this increased detection of ocular endothelial infection is due to the fact that the location and nature of the eye allows for detection of EHV-1 chorioretinopathy in the absence of clinical ocular disease, in contrast to the spinal cord, where detection of subtle lesions can be clinically inapparent and go undetected. Our study also provides evidence that the route and pathogenesis of EHV-1 infection of the endothelia of the spinal cord and the eye is similar, making the ocular model attractive for testing future vaccines or therapeutics in an immunologically relevant age group. Finally, there is evidence that EHV-1 infection primarily affects the choroidal vasculature, which would explain why lesions are not detectable in vivo until at later times after infection. Further examination of naturally occurring lesions in conjunction with EHV-1 as well as long term clinical relevance will be critical for an understanding of EHV-1 pathogenesis in the vascular endothelium and long-term studies may be important in the context of accurate prognosis following EHV-1 infection.

## Competing interests

The authors declare that they have no competing interests.

## Authors’ contributions

GSH, DPL and JS provided the funding for the outlined experiments. GSH prepared the manuscript, provided supervision for experiments 2 and 3 and performed data analysis. LSG provided clinical expertise and animal care for experiments 2 and 3 and performed the post mortem examination and histopathology on horse 11 in experiment 2. SBH performed the animal experiments and all clinical sampling for experiments 2 and 3 and performed FP, FFP and FA in experiment 3. NO and TH generated the Ab4GFP virus used in experiment 3. CP provided expertise with ophthalmological exams and use of the fundus camera and the FA camera. Jesse Hand performed FA in experiment 2 and provided training with the camera. CH performed growth curves on parental and mutant viruses. JS conducted experiment 1, and helped with the design and the evaluation of experiments 2 and 3. All authors read and approved the final manuscript.

## Supplementary Material

Additional file 1 **Generation of mutant viruses.** a. Replacement of ORF75 of EHV-1 strain Ab4 by the LacZ gene resulting in AB4Δ 75-LacZ, see also Sun et al. [13]. b. Insertion of green fluorescent protein between the ORF1 and ORF2 genes of EHV-1 strain Ab4 using *en passant* recombination resulting in Ab4GFP. Click here for file

Additional file 2 **In vitro growth characteristics of the Ab4 WT and ΔORF1/2 viruses.** Titers were measured on RK-13 cells and represent results of 3 repeats. Ab4 WT virus are represented as squares, Ab4GFP are represented as diamonds. Intracellular virus titers (a) and extracellular viral titers (b) are depicted. Data are displayed as means ± STDEV. Click here for file

Additional file 3 **Detection of LacZ expression in tissues collected post mortem following infection of ponies with Ab4∆75-LacZ (experiment 1).** For detection of beta galactosidase activity, tissues were collected from ponies at different times as indicated in the table and stained with X-gal. Positive LacZ expression indicates presence of virus in the respective tissues. Click here for file
