# Supplementary figures and images for: Experimental infection with equine herpesvirus type 1 (EHV-1) induces chorioretinal lesions
Source: Vet Res. 2013 Dec 5;44(1):118. doi: 10.1186/1297-9716-44-118 (PMC4028784; doi:10.1186/1297-9716-44-118)

a.

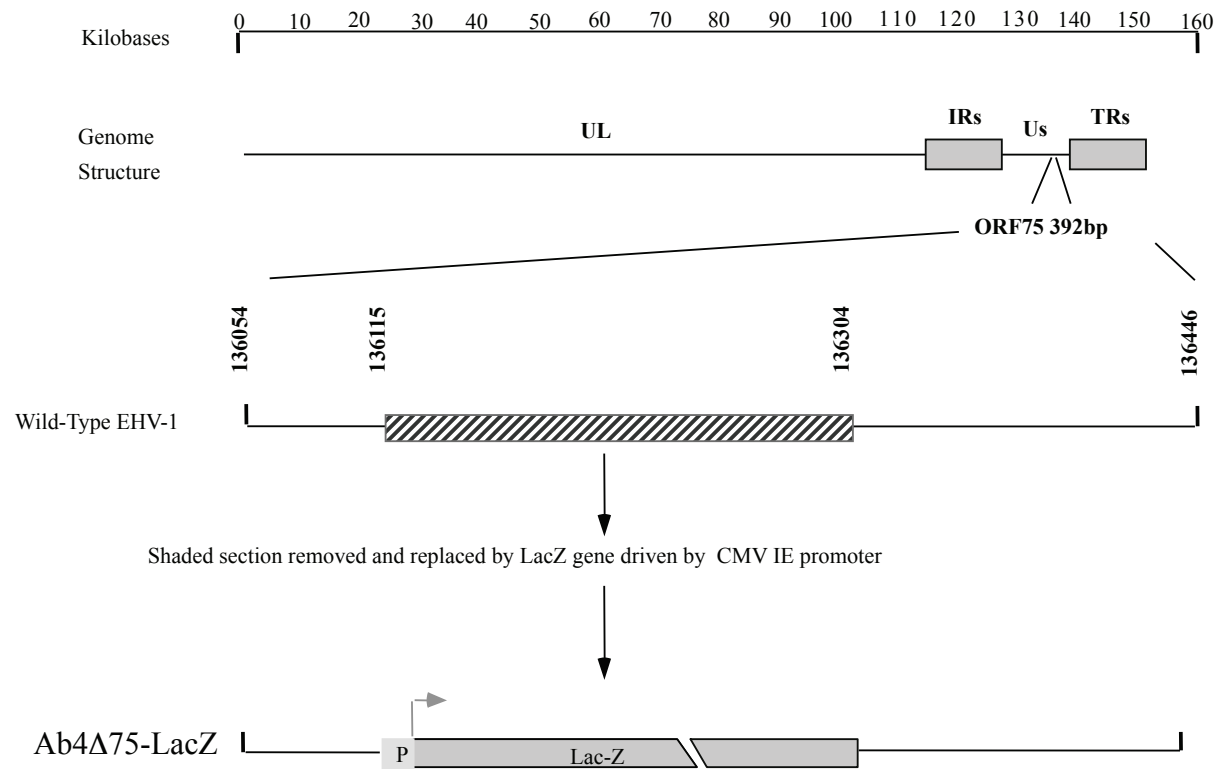

b.

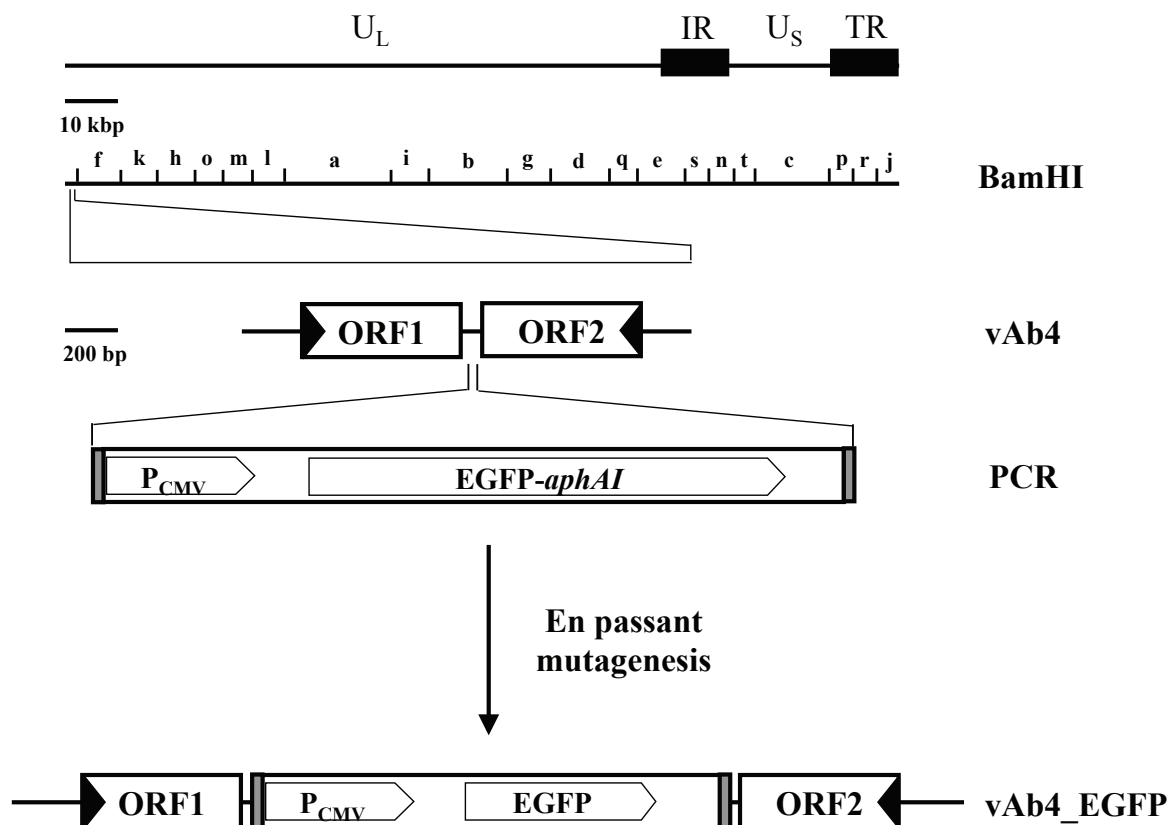

Supplement: Additional file 1 — Generation of mutant viruses. a. Replacement of ORF75 of EHV-1 strain Ab4 by the LacZ gene resulting in AB4Δ 75-LacZ, see also Sun et al. [13]. b. Insertion of green fluorescent protein between the ORF1 and ORF2 genes of EHV-1 strain Ab4 using en passant recombination resulting in Ab4GFP. [file 1297-9716-44-118-S1.pdf]

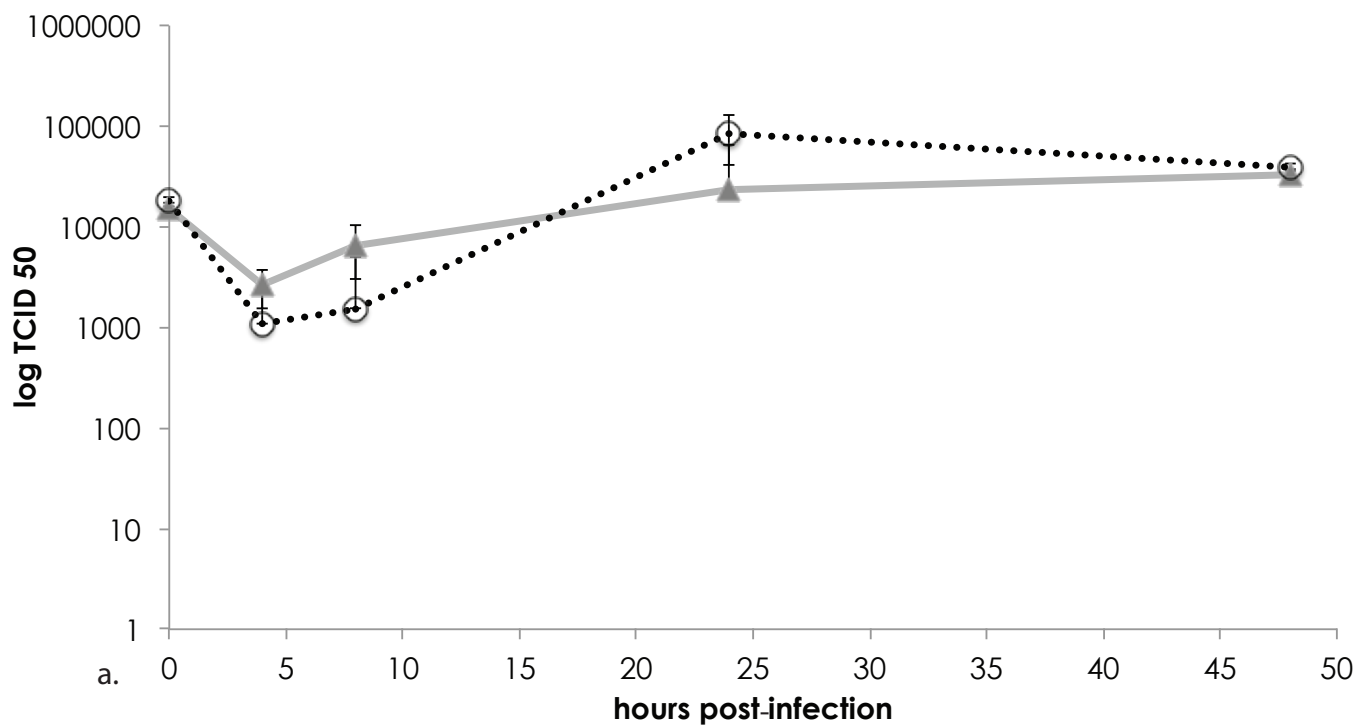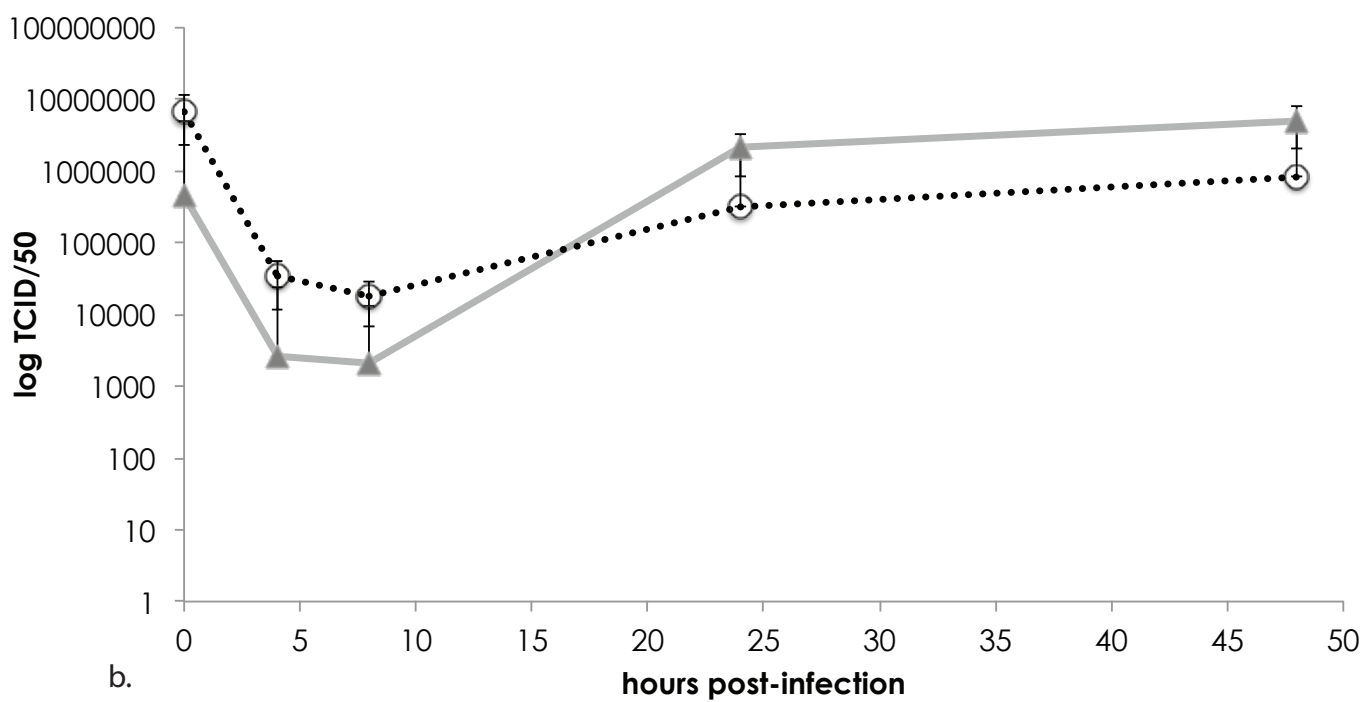

Supplement: Additional file 2 — In vitro growth characteristics of the Ab4 WT and ΔORF1/2 viruses. Titers were measured on RK-13 cells and represent results of 3 repeats. Ab4 WT virus are represented as squares, Ab4GFP are represented as diamonds. Intracellular virus titers (a) and extracellular viral titers (b) are depicted. Data are displayed as means ± STDEV. [file 1297-9716-44-118-S2.pdf]
