# Supplementary material for: Experimental infection with equine herpesvirus type 1 (EHV-1) induces chorioretinal lesions
Source: Vet Res. 2013 Dec 5;44(1):118. doi: 10.1186/1297-9716-44-118 (PMC4028784; doi:10.1186/1297-9716-44-118)
Supplement: Additional file 3 — Detection of LacZ expression in tissues collected post mortem following infection of ponies with Ab4∆75-LacZ (experiment 1). For detection of beta galactosidase activity, tissues were collected from ponies at different times as indicated in the table and stained with X-gal. Positive LacZ expression indicates presence of virus in the respective tissues. [file 1297-9716-44-118-S3.docx]

| **Pony #** | | A | B | C | D | E | F | G | H |
| --- | --- | --- | --- | --- | --- | --- | --- | --- | --- |
| **Tissue** | **Day pi/euthanasia** | 1 | 2 | 3 | 5 | 9 | 12 | 19 | 23 |
| Trachea | | + | - | + | - | - | - | - | - |
| Lung | | + | + | - | - | - | - | - | - |
| Submandibular lymph node | | + | + | + | + | + | - | - | - |
| Retropharyngeal lymph node | | - | + | + | + | - | - | - | - |
| Bronchial lymph node | | - | - | + | + | - | - | - | - |
| Mediastinal lymph node | | - | - | - | - | - | - | - | - |
| Trigeminal ganglion | | - | + | + | - | - | - | - | - |
| Chorioretinal endothelium | | - | - | - | - | + | - | - | - |

+ LacZ expression as indicated by X-gal positive tissues; - no LacZ expression.
